# Effects of Cognitive-Behavioral Therapy on Psychological Resilience, Social Adaptation and Clinical Efficacy of Patients with Bone Tumors

**DOI:** 10.12669/pjms.39.4.7279

**Published:** 2023

**Authors:** Lei Gao, Weina Yu, Ce Wang

**Affiliations:** 1 Lei Gao, Department of Orthopedics, Baoding No.1, Central Hospital, Baoding 071000, Hebei, China; 2 Weina Yu, Department of Orthopedics, Baoding No.1, Central Hospital, Baoding 071000, Hebei, China; 3 Ce Wang, Department of Medical Oncology, Affiliated Hospital of Hebei University, Baoding 071000, Hebei, China

**Keywords:** Cognitive-Behavioral Therapy, Bone Tumor, Psychological Resilience, Social Adaptation, Treatment

## Abstract

**Objective:**

To evaluate the effects of cognitive-behavioral therapy on psychological resilience, social adaptation and clinical efficacy in patients with bone tumors.

**Methods:**

This is a retrospective study. Eighty patients with bone tumor admitted to Baoding No.1 Central Hospital were included and randomly divided into two groups: the experimental group and the control group, with 40 cases in each group from March 2020 to February 2022. Patients in the control group were given conventional specialist care, while those in the experimental group were given cognitive-behavioral therapy on top of the treatment in the control group. The differences in quality of life before and after treatment between the two groups were compared and analyzed.

**Results:**

The levels of SAS and SDS were significantly lower in the experimental group compared to the control group, with statistically significant differences (p<0.05). The satisfaction level in the experimental group was higher than in the control group, with statistically significant difference (p=0.04). In addition, the psychological resilience scores of adaptability, toughness, control and goal achievement in the experimental group were significantly improved compared with those in the control group, with statistically significant differences (p<0.05); The cognitive scores in the experimental group were significantly higher than those in the control group, with statistically significant difference (p<0.05).

**Conclusion:**

Cognitive-behavioral therapy is an effective regimen for patients suffering from bone tumors, boasting various benefits such as significantly enhanced patient compliance with treatment, improved quality of life, increased resilience, ameliorated anxiety and depressive states, and improved treatment efficacy and patient satisfaction.

## INTRODUCTION

With the increasing aging of society and changes in lifestyle and quality of life, malignant bone tumors have witnessed an increasing incidence.^1^ Bone tumor is a serious orthopedic disease that poses a great danger to patients. Usually, patients mostly develop bone tumors in the lower extremities, mainly in the proximal tibia, distal femur, and proximal humerus epiphysis.^2^ The tumor cells directly form bone-like tissue, and the tumor may involve the periosteum, bone cortex and medullary cavity, with joint and bone pain and local masses as the main manifestations. Malignant bone tumors are characterized by insidious onset, rapid disease progression, and extremely high disability and mortality rates, which seriously affect the quality of life and overall survival of patients.^3^

Clinically, malignant bone tumors are mainly treated with comprehensive treatment methods, including early surgical treatment and implementation of chemotherapy before and after surgery. Patients with bone tumors usually have a long treatment and recovery period, during which they are prone to negative and pessimistic emotions, which are not conducive to the development of all treatments and nursing care, resulting in poor prognosis. Especially, radiotherapy and other chemotherapy will not only lead to physiological adverse reactions such as nausea, vomiting, hair loss and pain, but also negatively affect their psychological behaviors such as cognitive function, emotional control and disease coping style.^4^

Negative emotion is a kind of mental depression that, in physiological aspect, leads to endocrine dysregulation and lower immunity of cancer patients, which worsens their condition.^5^ And in the psychological aspect, it leads to resistance to treatment, and in serious cases, suicidal tendencies, threatening life safety. It has been shown in a study^6^ that patients have disease cognitive biases about cancer during treatment, leading to their low psychological resilience and high incidence of negative emotions, which affect their physical and psychological recovery and long-term survival quality. Cognitive-behavioral therapy is a psychological treatment method that effectively corrects cognitive biases by means of cognitive reconstruction and thus eliminates undesirable emotions and behaviors.

It is a common clinical psychological intervention that uses cognitive assessment, correct cognitive reconstruction and rational cognitive intervention to correct patients’ misperceptions, help them increase their self-management knowledge and improve their coping behaviors.^7^,^8^ In this study, cognitive-behavioral therapy was performed on patients with bone tumors, showing certain success in terms of psychological resilience, social adaptation and clinical efficacy.

## METHODS

This is a retrospective study. Eighty patients with bone tumor admitted to Baoding No.1 Central Hospital were recruited and randomly divided into two groups: the experimental group and the control group, with 40 cases in each group from March 2020 to February 2022. In the experimental group, there were 26 males and 14 females, aged 28-70 years, with an average of 63.85 ± 7.06 years; In the control group, there were 23 males and 17 females, aged 27-70 years, with an average of 63.46 ± 7.14 years. No significant difference was observed in the comparison of general data between the two groups, which was comparable (Table-I). The study was approved by the Institutional Ethics Committee of Baoding No.1 Central Hospital(No.:HDFYLL-KY-2022-006; date: October 15,2022), and written informed consent was obtained from all participants.

**Table-I:** Comparative analysis of the general data of the experimental group and the control group (*χ̅*±*S*) n=40.

Indicator	Experimental group	Control group	t/χ^2^	P
Age (years)	63.85±7.06	63.46±7.14	0.38	0.70
Male (cases %)	26 (65%)	23 (57.5%)	0.47	0.49
***Lesion site***				
Femur (cases %)	17 (42.5%)	19 (47.5%)	0.20	0.65
Tibia (cases %)	13 (32.5%)	16 (40%)	0.49	0.49
Humerus (cases %)	10 (2.5%)	5 (12.5%)	2.05	0.15
***Type of pathology***				
Osteosarcoma (cases %)	21 (52.5%)	20 (50%)	0.05	0.82
Chondrosarcoma (cases %)	9 (22.5%)	11 (27.5%)	0.27	0.61
Fibrosarcoma (cases %)	3 (7.5%)	2 (5%)	0.21	0.64
Giant cell tumor of bone (cases %)	4 (10%)	2 (5%)	0.72	0.40
Other (cases %)	3 (7.5%)	5 (12.5%)	0.56	0.47
Duration of disease (years)	2.06±0.72	2.10±0.83	0.23	0.82

p>0.05.

### Inclusion criteria:

- Patients who met the diagnostic criteria of bone tumors.^9^
- Patients who all obtained pathological results through biopsy.
- Patients aged <70 years.
- Patients with an expected survival period greater than 12 months.
- Patients who were conscious, free of mental disorders, and able to actively cooperate with the implementation of treatment and care plans.
- Patients with complete clinical data.

### Exclusion criteria:

- Patients with serious heart, liver, lung, kidney, brain and other organ disorders.
- Patients with serious complications such as fractures.
- Patients with combined malignant tumors of other sites.
- Patients with severe osteoporosis at the same time.
- Patients with expected survival less than 12 months.
- Patients with severe psychiatric disorders and unable to satisfactorily cooperate with the study.

Patients in the control group were given routine specialist care, i.e., completion of care according to medical prescriptions, issuance of health handbooks, lectures on bone tumors, individualized guidance and regular follow-up visits. Patients were instructed to eat a high protein and vitamin diet to strengthen their resistance and to take medication according to medical prescriptions.

In contrast, those in the experimental group were given cognitive behavioral therapy (CBT) on top of the control group. First, experienced nursing staff with specialized care were selected for the implementation of CBT, which began after the patient was admitted to the hospital and continued until the patient was discharged. The main therapeutic steps are as follows:

### Cognitive reconstruction:

a quiet ward or small meeting room was chosen to communicate with the patient alone, and the patient was guided by professional psychological methods in order to as much as possible the real inner thoughts and the perceptions about the disease, therapy, prognosis, post-operative self-image and social interaction. Correction was made against the patient’s wrong, negative and unfavorable perception of treatment and recovery.

If the patient believes that he/she has advanced tumor and the prognosis is poor even with surgery and will end up with no money, the nursing staff should inform the patient that with the development of medical technology, the prognosis of bone tumor has been significantly improved and he/she can still return to his/her original job after active treatment and rehabilitation. Eventually, patient confidence and hope levels were raised by presentations of successful treatment cases.

### Explanation of relevant knowledge:

Based on the patients’ education level and social background, the means that patients can accept and understand were used, such as dialogue and communication, picture display, showing video, etc., to explain the occurrence of bone tumor, the choice of therapeutic methods, the advantages and disadvantages of each means, and the way of recovery. As a result, patients can accept the fact of illness with an objective and peaceful attitude, and actively adjust their mindset to face the disease and establish the belief of overcoming the disease.

### Behavioral therapy:

Patients were instructed to perform rehabilitation exercises, take reasonable rest and assist with exercise.

### Family and social support:

Some patients’ families often develop anxiety, entanglement, and even a sense of illness shame after being informed of the patients’ conditions, which can further lower the patients’ level of hope and lead them to adopt a negative attitude toward the disease.

Nursing staff should carry out health education to the family members of the patients by adopting separate communication and centralized teaching, so that they can take a correct attitude towards towards the patients’ conditions, such as frequent communication with the patients. In this way, patients will feel valued and cared for by their families and society. Furthermore, patients should be encouraged to take the initiative to seek support and help, talk more with family, friends and health care workers to reduce psychological stress, and participate in more recreational activities to improve social adaptation.

### Observation indicators:

Comparative analysis of the quality of life of the two groups before and after therapy: The Generic Quality of Life Inventory-74 (GQOLI-74) was used to evaluate the two groups in terms of physical function, psychological function, social function, and material life status. The questionnaire has a total of 20 questions and 74 items, and each it

### Comparative analysis of emotional status:

Self-rating Anxiety Scale (SAS) and Self-rating Depression Scale (SDS)^11^ were used to assess the emotional change of the two groups before and after therapy, respectively, with lower scores indicating better emotional status.

### Comparative analysis of patient satisfaction:

The Patient Satisfaction Questionnaire Short Form (PSQ-18) was used^12^ for comparative analysis of patient satisfaction before and after therapy, including very satisfied, relatively satisfied, satisfied, uncertain, and unsatisfied, with total satisfaction = (very satisfied + relatively satisfied + satisfied)/total number of cases x 100%. Comparative analysis of psychological resilience before and after therapy in two groups.

The Connor-Davidson Resilience Scale (CD-RISC) was used to assess the psychological resilience of the two groups before and after therapy, involving factors such as adaptability, toughness, control and goal achievement.The scale contains 25 items with a total score range of 0-100, with higher scores indicating better psychological resilience.

### Comparative analysis of cognitive level before and after therapy:

The cognitive level was assessed by a self-administered scale, including basic knowledge of the disease, precautions for rehabilitation exercises, daily diet and precautions during postoperative and radiotherapy. The total score was 100, and the higher the score, the higher the cognitive level.

### Statistical analysis:

All data in this study were statistically analyzed by SPSS 20.0 software, and measurement data were expressed as (*χ̅*±S). Two independent sample t-test was used for comparison between groups, paired t test was used to analyze data within groups, and χ^2^ test was used for the comparison of rates. P<0.05 indicates a statistically significant difference.

## RESULTS

Before cognitive-behavioral therapy, no statistically significant difference was observed between the two groups in the scores of physical function, psychological function, social function and material life status (p>0.05). After therapy, patients in the experimental group showed significant improvement in the above indicators compared to the control group, with statistically significant differences (p<0.05) (Table-II).

**Table-II:** Comparative analysis of quality of life scores before and after therapy in the two groups (*χ̅*±*S*) n=40.

Indicator		Experimental group	Control group	t	p
Physical function	Before therapy	42.58 ± 7.63	42.37 ± 7.51	0.12	0.90
	After therapy*	55.47 ± 7.08	51.36 ± 7.87	2.46	0.02
Psychological function	Before therapy	48.50 ± 6.53	48.16 ± 6.77	0.23	0.82
	After therapy*	54.49 ± 7.24	50.43 ± 7.32	2.49	0.01
Social function	Before therapy	60.23 ± 8.59	61.04 ± 8.62	0.42	0.67
	After therapy*	68.27 ± 7.42	63.97 ± 7.82	2.52	0.01
Material life status	Before therapy	42.80 ± 6.15	42.57 ± 6.43	0.16	0.87
	After therapy*	54.39 ± 6.27	50.18 ± 6.07	3.05	0.00

* p<0.05.

Before cognitive-behavioral therapy, no statistically significant difference was observed between the two groups in the levels of SAS and SDS (p>0.05). After therapy, the levels of SAS and SDS were significantly lower in the experimental group compared to the control group, with statistically significant differences (p<0.05) (Table-III). The satisfaction level in the experimental group was 95%, which was significantly higher than 80% in the control group, with a statistically significant difference (p=0.04) (Table-IV).

**Table-III:** Comparative analysis of emotional state before and after therapy in the two groups (*χ̅*±*S*) n=55.

Indicator		Experimental group*	Control group	t	p
SAS	Before therapy	61.58 ± 6.41	61.80 ± 6.55	0.15	0.88
	After therapy*	51.17 ± 5.21	55.63 ± 6.25	3.47	0.00
SDS	Before therapy	63.24 ± 6.58	63.17 ± 6.36	0.05	0.96
	After therapy*	54.57 ± 7.25	50.46 ± 7.30	2.53	0.01

* p<0.05.

**Table-IV:** Comparative analysis of patient satisfaction between the two groups (*χ̅*±*S*) n=40.

Group	Very satisfied	Relatively satisfied	Satisfied	Uncertain	Dissatisfied	Total satisfaction*
Experimental group	30	6	2	0	0	38 (95%)
Control group	21	8	3	1	7	32 (80%)
χ^2^						4.11
P						0.04

* p<0.05.

Before cognitive-behavioral therapy, no statistically significant difference was observed between the two groups in the psychological resilience scores of adaptability, toughness, control and goal achievement (p>0.05). After therapy, the above indicators in the experimental group were significantly improved compared with those in the control group, with statistically significant differences (p<0.05) (Table-V).

**Table-V:** Comparative analysis of psychological resilience of the two groups before and after therapy (*χ̅*±*S*) n=40.

Indicator		Experimental group	Control group	t	p
Adaptability	Before therapy	13.86 ± 2.53	13.73 ± 2.55	0.23	0.82
	After therapy*	21.48 ± 3.42	15.31 ± 3.34	12.13	0.00
Toughness	Before therapy	12.63 ± 1.54	12.49 ± 1.70	0.38	0.70
	After therapy*	19.64 ± 2.28	14.57 ± 2.12	10.30	0.00
Control	Before therapy	13.27 ± 2.56	13.04 ± 2.60	0.40	0.69
	After therapy*	22.05 ± 2.12	16.32 ± 2.18	11.92	0.00
Goal achievement	Before therapy	11.82 ± 1.25	12.03 ± 1.13	0.79	0.43
	After therapy*	21.30 ± 3.17	16.31 ± 3.05	7.17	0.00

* p<0.05.

Before cognitive-behavioral therapy, no statistically significant difference was observed between the two groups in the cognitive scores (p>0.05). After therapy, the cognitive scores in the experimental group were significantly higher than those in the control group, with a statistically significant difference (p<0.05) (Table-VI).

**Table-VI:** Comparative analysis of the cognitive scores of the two groups before and after therapy (*χ̅*±*S*) n=40.

Group	Before therapy	After therapy*
Experimental group	24.56 ± 4.57	89.73 ± 10.67
Control group	24.73 ± 3.86	65.92 ± 9.53
*t*	0.18	10.36
*P*	0.86	0.00

* p<0.05.

## DISCUSSION

It was confirmed in our study that patients in the experimental group had significantly better somatic, psychological, social and material life status scores after receiving the cognitive-behavioral therapy compared to the control group (p<0.05); After therapy, the levels of SAS and SDS were significantly lower in the experimental group compared with the control group (p<0.05); The satisfaction level in the experimental group was 95%, which was significantly higher than 80% in the control group (p=0.04). All of these indicate that cognitive-behavioral therapy boasts effective improvement of anxiety and depression in patients with bone tumor, and thus plays a negative role in relieving bad mood. Meanwhile, appropriate exercise is beneficial to improve the immune function of the body and relax the mind, which can regulate the physical and mental health.

Bone tumor, a malignant neoplastic disease occurring in bone tissue, manifests as bone deposition and destructive bone resorption.^13^ Rapid progression of bone tumors may cause pain, swelling and dysfunction, affecting limb activities; in severe cases, it leads to pathological fractures, which seriously affects patients’ daily life.^14^ Clinically, surgery and chemotherapy are commonly used as methods to control the growth and metastasis of malignant bone tumors. However, such therapies are accompanied by postoperative pain, chemical cytotoxicity, and increase toxic side effects such as nausea and vomiting. This, in turn, leads to reduced immunity, adverse emotions such as anxiety or depression, and seriously affects the quality of survival.^15^ Given factors such as inadequate social support, disease progression and metastasis, patients with tumors often respond to the disease with negative emotions, which are the main risk factors for reducing clinical outcomes and affecting prognosis.^16^

Patients with bone tumors suffer from both physical and mental pain as well as delayed recovery process due to longer treatment period, adverse reactions during treatment, and negative emotions formation.^17^ Cancer is a stressor resulting in a high level of emotional and cognitive impairment in patients suffering from it, which is attributed to diagnosis-related distress, impairment of quality of life and side effects of primary treatment.^18^ Such side effects may have a serious impact on the mental health of patients. It was noted that half of the patients with chronic diseases and cancer have fear of disease recurrence at moderate to high levels.^19^ Patients may feel nervous, fearful, uneasy, doubtful, anxious and other discomfort when they are first admitted to the hospital in the face of unfamiliar environment, unknown treatment effect and prognosis. After surgery, patients are temporarily unable to move their limbs due to the characteristics of bone tumors, and there are no recreational activities during hospitalization.

This leads to a number of negative consequences, such as patients focusing on their own disease and treatment, worrying about pathological results, economic costs of treatment and rehabilitation effects, etc., and are prone to negative emotional experience. Complemented by patients’ uncertainty and unreasonable cognition about their disease, they also weaken their treatment confidence and are in a negative state and lack of treatment motivation, which is not conducive to patients’ disease regression.^20^ For this reason, rational care measures are important in reducing the impact of adverse reactions and negative emotions on patients’ recovery from treatment and helping them to get rid of their physical activity disorders as early as possible.

Cognitive behavioral therapy is an active, problem-focused collaboration with patients.^21^ It is a common clinical psychological intervention that uses cognitive assessment, correct cognitive reconstruction and rational cognitive intervention to correct patients’ misperceptions, help them increase their self-management knowledge and improve their coping behaviors. Cognitive-behavioral therapy enables nursing staff to help patients identify, assess, and modify inaccurate or otherwise unhelpful thinking related to emotional distress^22^ so that their thinking is consistent with that of medical personnel.

Psychological resilience refers to the ability of an individual to recover to its original state after being subjected to stressful events and trauma. Generally, good psychological resilience is beneficial for patients to establish recovery confidence.^23^ Psychological resilience, as a positive psychological trait of human beings, promote individuals to maintain a high psychological adaptability in times of adversity. It was suggested in our study that after the cognitive-behavioral therapy, the psychological resilience scores of the experimental group, such as adaptability, toughness, control, and goal achievement, were significantly improved compared with those in the control group, with statistically significant differences (p<0.05).

After therapy, the cognitive score of the experimental group was significantly higher than that of the control group, with a statistically significant difference (p<0.05). All of these indicate the effectiveness of the cognitive-behavioral therapy in improving the psychological resilience of patients with bone tumors. It is explained that the cognitive-behavioral therapy increases social support among patients, improves emotional function, and forms an optimistic and tenacious state of mind.^24^ It also increases the patients’ correct perception of their disease, which leads to positive acceptance of therapy. In the process of cognitive-behavioral therapy, patients’ confusion should be grasped through motivational interviews and answered patiently. Furthermore, patients should be organized to participate in health knowledge lectures and provided with health knowledge manuals at discharge, which can play a favourable role in cognitive reconstruction and improve their disease awareness.

### Limitations of the study:

It includes fewer observation cases were included, and the follow-up time. It will help to more objectively evaluate the advantages and disadvantages of the cognitive-behavioral therapy and benefit more patients.

## CONCLUSIONS

To put it in a nutshell, cognitive-behavioral therapy is an effective regimen for patients suffering from bone tumors, boasting various benefits such as improved patients’ correct cognition of the disease, ameliorated anxiety and depressive states, significantly enhanced patient compliance with treatment, ameliorated quality of life, and improved patient satisfaction. It is worthy of clinical promotion for its many benefits described.

### Authors’ Contributions:

**LG** and **WY:** performed the study, data collection, are responsible and accountable for the accuracy and integrity of the work;

**CW:** performed the statistical analysis and participated in its design;

**WY:** participated in acquisition, analysis, or interpretation of data and draft the manuscript. All authors read and approved the final manuscript.
